# Exploring Macrophage Activation Syndrome Secondary to Systemic Lupus Erythematosus in Adults: A Systematic Review of the Literature

**DOI:** 10.7759/cureus.18822

**Published:** 2021-10-16

**Authors:** Afia Aziz, Everardo E Castaneda, Noorain Ahmad, Harish Veerapalli, Amy G Rockferry, Chetan Reddy Lankala, Pousette Hamid

**Affiliations:** 1 Internal Medicine, California Institute of Behavioral Neurosciences & Psychology, Fairfield, USA; 2 Medicine, California Institute of Behavioral Neurosciences & Psychology, Fairfield, USA; 3 Neurology, California Institute of Behavioral Neurosciences & Psychology, Fairfield, USA

**Keywords:** rheumatic disorder, sle, systemic lupus erythematosus, secondary hemophagocytic lymphohistiocytosis, hlh, hemophagocytic lymphohistiocytosis, hemophagocytic syndromes, macrophage activation syndrome

## Abstract

Among the autoimmune (AI) diseases, systemic lupus erythematosus (SLE) is known to mimic various disease processes and this can lead to under-diagnosis of macrophage activation syndrome (a dire complication). We aimed at performing a systematic review to identify trigger factors that could lead to the development of macrophage activation syndrome (MAS) in patients with SLE as well as identify factors that can affect mortality. We searched the following databases to extract relevant articles: PubMed, ScienceDirect, Cochrane library, Pro-Quest, and Google Scholar. We used search terms including but not limited to hemophagocytic syndromes OR hemophagocytic lymphohistiocytosis OR macrophage activation syndrome OR HLH OR secondary hemophagocytic lymphohistiocytosis AND systemic lupus erythematosus OR SLE. We screened the articles first by titles and abstracts and later by full text. After the application of our eligibility criteria, we identified eight studies to include in our final synthesis. The studies showed that lupus flare itself, as well as, time to onset and high systemic lupus erythematosus disease activity index (SLEDAI) scores, were major risk factors that led to the development of MAS. In addition, infections followed by drugs, underlying malignancy, and pregnancy were other potential trigger factors identified. Studies also detected that MAS development led to high intensive care unit (ICU) admissions and in-hospital mortalities with C-reactive protein (CRP) levels, age, presence of infection, leukopenia, thrombocytopenia, MAS throughout the hospital stay, and high liver function tests (LFTs) as signs of poor prognosis. Additionally, ferritin levels, LFTs, and triglyceride levels formed an important part of diagnostic criteria. However, our review was limited due to the absence of prospective studies and heterogeneity in the studies seen. More studies need to be done to identify various factors leading to hemophagocytic lymphohistiocytosis (HLH) in autoimmune diseases with validated criteria for MAS secondary to autoimmune diseases.

## Introduction and background

Systemic lupus erythematosus (SLE) is a complex disease with a multitude of presentations and complications making it a notorious mimicker of other disease processes. In the United States, SLE has a prevalence of 73 per 100,000 person-years with women nine times more likely to be affected than men [1]. In addition, the disease is more common in individuals from Africa, followed by Asians and Hispanics, and least common in Caucasians [2]. The incidence of SLE has tripled in the last 40 years and survival rates have also drastically improved due to a combination of early recognition of mild disease and better interventions [3].

However, despite improvement in diagnostic and treatment approaches, there is still limited knowledge about one of its rarer known but devastating complications, namely macrophage activation syndrome (MAS). Macrophage activation syndrome's prevalence in SLE is thought to be between 0.9% and 4.6% [4]. Because of the similarity in clinical presentation, diagnosis of MAS in patients with SLE is often challenging leading to a somewhat under-representation of MAS in this population. Moreover, late diagnosis can also lead to increased morbidity and mortality in patients with SLE due to differences in therapeutic approaches. The standardized mortality rate in patients with SLE due to various causes has been estimated to be around 2.4% [5].

Macrophage activation syndrome is one of the many different types of hemophagocytic syndromes (HPS) described in the literature. It is a form of hemophagocytic lymphohistiocytosis (HLH) secondary to rheumatic diseases, which is characterized by the presence of hypercytokinemia leading to inflammation, and organ dysfunction which may progress to multi-organ failure. Diagnosis of MAS is complicated and often based on multiple criteria that have changed over the years, including the HLH-2004 clinical criteria which required at least the presence of molecular diagnosis consistent with HLH or five out of nine findings that include fever >38.5; splenomegaly; peripheral blood cytopenias (at least any two); hypertriglyceridemia; hemophagocytosis in either bone marrow, spleen, lymph node or liver; low or absent natural killer (NK) cell activity; hyperferritinemia; elevated soluble interleukin-2 receptor alpha chain (CD25) or elevated chemokine (C-X-C motif) ligand 9 (CXCL9).

Another set of criteria were laid by the European League Against Rheumatism (EULAR)/American College of Rheumatology (ACR)/Pediatric Rheumatology International Trials Organization (PRINTO) in 2016 for the diagnosis of MAS for juvenile idiopathic arthritis (JIA). Even though it has been used in different studies to identify MAS in autoimmune (AI) diseases and is more reliable in diagnosing MAS secondary to lupus than HLH-2004 criteria, its validation for its use in lupus is yet to be proved [6]. The 2016 criteria state that a patient with JIA is classified with MAS if the following criteria are met with fever, ferritin >684 ng/ml, and any two of the following: (i) platelet count </=181 x10^9^/L, (ii) aspartate transaminase (AST) >48 units/liter, (iii) triglycerides >156 mg/dl, and (iv) fibrinogen </=360 mg/dl.

In addition, a third criterion proposed by Fardet et al. comprises nine variables that are often used in adults with reactive HLH and is the only validated criteria in this population, though its use in MAS is still not validated [7].

Systemic lupus erythematosus has seen various changes in its diagnostic criteria over the years with the most recent one being the 2019 Joint European League against Rheumatism/ American College of Rheumatology (EULAR/ACR) criteria which were introduced to improve the sensitivity and specificity of early criteria and to also improve the detection of early-onset SLE [8]. Other criteria that have been used before were the 2012 Systemic Lupus International Collaborating Clinics (SLICC) and the 1997 American College of Rheumatology (ACR) criteria [9,10].

The pathogenesis of MAS is poorly understood but thought to be secondary to hyper-stimulation of macrophages and cytotoxic T lymphocytes (CD8+ T cells) which secrete a large number of cytokines resulting in a cytokine storm. Some cytokines that have been implicated in the pathogenesis are interleukin-2 (IL-2), interleukin-6 (IL-6), interleukin-18 (IL-18), and interferon-gamma (IFN-γ) [11]. On the other hand, despite improvement in diagnostic criteria of SLE, its pathophysiology is still a perplexing issue. The intricate pathogenesis of SLE has been widely studied over the years with various factors contributing to it, namely genetic, environmental, and immunologic. The major pathways identified are complement deficiencies, breakdown of self-tolerance with dysfunction of myeloid and lymphoid cells as well as imbalance in pro-inflammatory and anti-inflammatory cytokines, apoptosis dysregulation, and impaired clearance of nucleic acids in neutrophil extracellular traps (NETs) and apoptotic bodies. Moreover, the presence of elevated levels of type 1 interferon (IFN) is a hallmark of SLE [12].

Macrophage activation syndrome is most commonly associated with juvenile idiopathic arthritis and in general, little is known about the association of MAS in SLE despite a large number of case reports and case series reported both in adult and pediatric populations. Our review aims at identifying characteristics of patients with SLE and MAS while identifying potential risk factors contributing to MAS in SLE as well as predisposing factors that contribute to the mortality associated with MAS secondary to SLE. Identifying various factors that are associated with triggers of MAS in SLE, as well as the potential risk factors contributing to increased mortality rates in this patient group, will help in categorizing patients at high risk of adverse outcomes. Risk stratification in such patients will also improve the prognosis by early identification of disease and timely treatment.

## Review

Methodology

Search Strategy

The literature search strategies, inclusion and exclusion criteria were conducted as per the Preferred Reporting Items for Systematic Reviews and Meta-Analyses (PRISMA) checklist 2020 [13].

We searched five databases systematically: PubMed, ScienceDirect, Cochrane library, ProQuest, and Google Scholar. We started searching on June 6, 2021, and concluded our search on June 26, 2021. We used the following keywords in varying combinations: hemophagocytic syndromes OR hemophagocytic lymphohistiocytosis OR macrophage activation syndrome OR HLH OR secondary hemophagocytic lymphohistiocytosis AND systemic lupus erythematosus OR SLE. The full list of keywords and results generated is listed separately in the appendices (see Appendix A). We also used medical subject headings (MeSH) terms related to macrophage activation syndrome, hemophagocytic lymphohistiocytosis, and systemic lupus erythematosus in PubMed. And after initial screening yielded 7494 articles, these articles were then screened for duplicates removal as well as titles and abstract screening which narrowed the studies to 301 articles. These articles were then subjected to full-text screening by two authors independently. Any discrepancy in article selection was clarified by mutual discussion.

Eligibility Criteria

All adult patients with SLE who had at least one episode of HLH were included in the review. All articles that were peer-reviewed and were in free full-text in the English language were included. Only articles from January 2001 to May 2021 were included. All cross-sectional cohort studies and trials were included in the study to identify potential risk factors leading to HLH in patients with SLE and to identify mortality risk factors and outcomes. We also used descriptive studies to identify specific features of the adult lupus population who suffered from MAS.

We excluded all patients with SLE without HLH development. For this review, we also excluded the pediatric population. All editorials, case reports, and animal studies were omitted. Furthermore, we also excluded all articles that were not published in the last 20 years, were in languages other than English, were unpublished, and articles in gray literature. We removed all studies in which mortality outcomes or risk factors leading to HLH in SLE were not specified.

Quality Assessment

**Figure 1:**
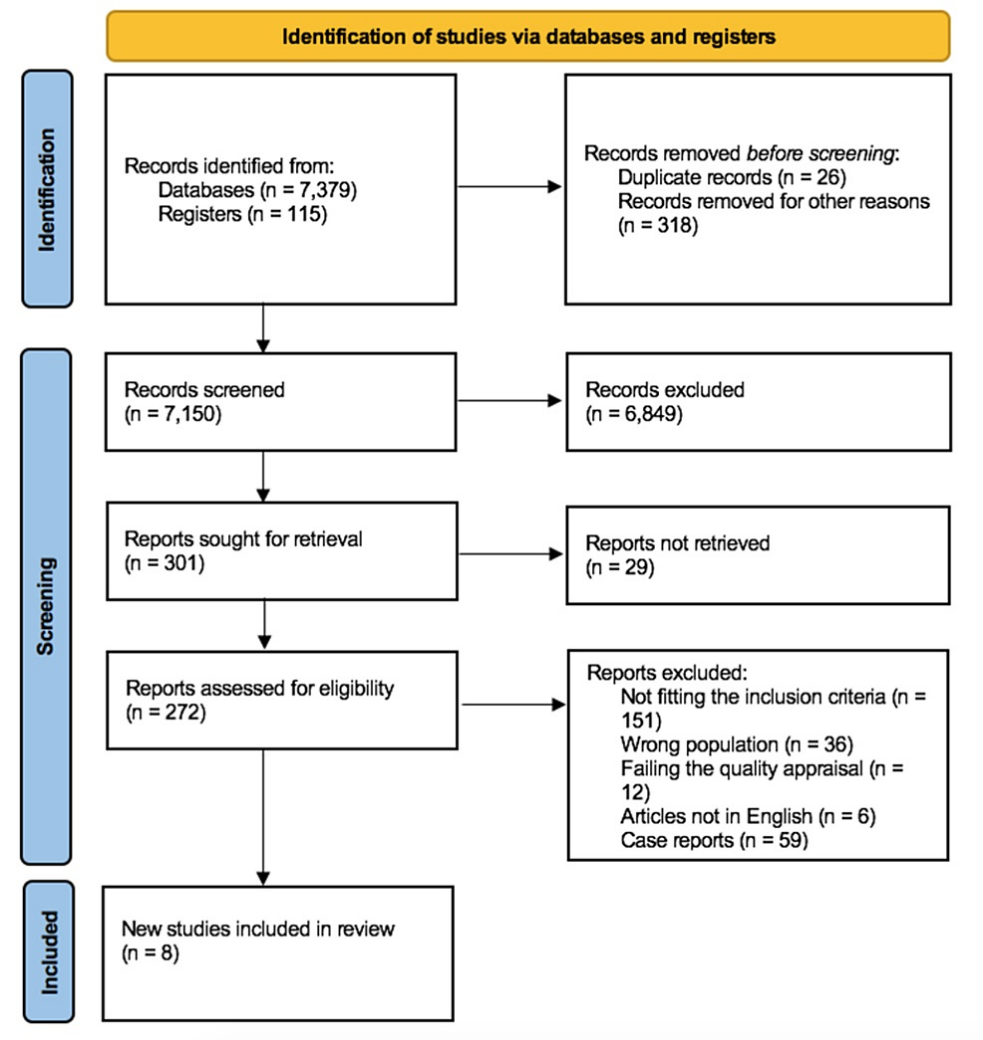
PRISMA flow diagram PRISMA = Preferred Reporting Items for Systematic Reviews and Meta-Analyses

After the removal of irrelevant articles and articles not fitting the inclusion criteria, we identified 22 studies for quality appraisal. All studies were retrospective. Out of which five were case-control studies and 15 cross-sectional studies with the remainder being case series. These articles were then assessed via the Modified Newcastle Ottawa Scale for case-control and observational studies while case series were identified via the National Institutes of Health (NIH) quality assessment tool. The PRISMA flow diagram depicting our search methodology is shown in Figure 1.

Results

Out of the 7150 records that were screened initially by titles and abstracts, only 272 reports were subjected to full-text review. The remaining were excluded mainly due to irrelevance to the topic, articles that were not in the English language or were not retrievable. Out of the 272 studies identified, we excluded all case reports and articles that did not fulfill the inclusion criteria. This led to a total of 22 articles which were then subjected to quality appraisal by using the Newcastle-Ottawa Scale for observational and case-control studies and the NIH assessment tool for case series. We deviated from our original intent to include case series as most of them had descriptions of other autoimmune disorders and diverse presentations. Finally, eight high-quality articles were subjected to the final review. Quality appraisal of case-control and cross-sectional studies used in our review via the Modified Newcastle-Ottawa Scale is shown in Table 1 and Table 2.

**Table 1:** Modified New-Castle Ottawa Scale for case-control studies ★: Star represents one point given to a study if it fulfills the criteria. Blank space shows that the study didn't fulfill the criteria. *Acceptable criteria for assessment ‘Selection’ of a study was its ability to define cases and controls based on independent validation, or records. ‘Cohort Representation’ means that the study is representative of an average patient with SLE in the community, and was given no score if selection bias was present. ‘Selection of Controls’ is from a hospital or community. 'Definition of Controls' should have SLE without MAS. **Acceptable criteria for assessment of ‘Comparability’ of a study is the presence of at least one confounder ‘age’  was controlled, resulting in that study to be awarded one point and two points if more than one confounder was controlled. ***Acceptable criteria for assessment of 'Outcome' were based on validation and records. With a point awarded if present, and another point awarded if the same method was used to ascertain cases and controls. 'Non-response Rates' that were explained received one point. And if no proper explanation was given, one point was deducted.

Study	Selection*				Comparability**		Outcome***			Total Score (9)	Quality of Evidence
	Cases Defined Adequately	Cohort Represent -ation	Selection of Controls	Definition of Controls	Control of At Least One Confounder	Control of Two or More Confounders	Ascertainment of Exposure	Same Method of Ascertainment for Cases and Controls	Non-response Rate		
Cohen et al. [14]	★		★	★	★	★	★	★		7	High
Huang et al. [15]	★	★	★	★	★	★	★	★	★	9	High
Ahn et al. [16]	★		★	★	★	★	★	★	★	8	High

**Table 2:** Modified New-Castle Ottawa Scale for cross-sectional studies * Acceptable criteria for assessment of ‘Selection’ of a study was based on four points with the fulfillment of each point resulting in one star. If the study selected patients that were representative or somewhat representative of patients with SLE having MAS, one point was awarded. And if no representation or description was present, a point was deducted. Points were awarded if the study had an adequate sample size with a diagnosis of MAS made with an assessment tool and a description of the assessment tool was present. Points were deducted if the sample size was small and diagnostic criteria for MAS in patients with SLE were not clear. ** 'Comparability' of a study was assessed if the study had controlled at least one confounder for which, one point was awarded. Two points were awarded if the study controlled additional factors. *** 'Outcome' of a study was assessed if there was the presence of blind assessment or record linkage for which, two points were awarded. And no points were awarded for self-reports. One point was awarded for the presence of an adequate statistical test.

Study	Selection*				Comparability**		Outcome***			Total Score	Quality of Evidence
	Representativeness of Exposed Cohort	Selection of Sample Size	Ascertainment of Exposure	Non-responders	Controlled for At Least One Confounder	Controlled for Two or More Confounders	Assessment of Outcome a) Independent Blind Assessment b) Record Linkage c) Self Report		Statistical Test		
Dallara et al. [17]	★		★	★	★		★	★	★	7	High
Takahashi et al. [18]	★		★	★	★		★		★	6	Fair
Fukaya et al. [19]	★	★	★	★	★		★	★	★	8	High
Lambotte et al. [20]	★	★	★	★	★		★	★		7	High
Gavand et al. [21]	★	★	★	★	★		★	★	★	8	High

These studies (five observational and three case-control studies) included a total pool of 249 patients with SLE who developed a total of 266 MAS episodes, the largest ever studied. We extracted data in terms of mortality and outcomes, patient trigger factors that may have contributed to the development of HLH as well as specific characteristics associated with SLE and MAS such as lab parameters like hemoglobin, platelets, white blood cells, ferritin, triglycerides, erythrocyte sedimentation rate (ESR), C-reactive protein (CRP), lactate dehydrogenase (LDH), aspartate aminotransferase (AST), alanine transaminase (ALT), fibrinogen, complement component 3 (C3), complement component 4 (C4), antinuclear antibody (ANA), anti-double stranded DNA (anti-dsDNA) levels as well as organ/system involvement like renal, pulmonary, serositis, cardiac, nervous and hematological.

Features, mortality outcomes, and trigger factors of studies included in our review are highlighted in Table 3 and Table 4.

**Table 3:** Table of features and outcomes of studies included in the systematic review CRP = C-reactive protein, LFTS = liver function tests, RFT = renal function tests, Hb = hemoglobin

No.	Author	Year	Country	Type of Study	Population	Sample Size	Follow-up Duration	Outcomes of Mortality
1.	Cohen et al. [14]	2018	USA	Case-control study	Adults	23	-	The in-hospital mortality of SLE with MAS patients was 19% as compared to 3% in SLE without MAS. High SLEDAI values were associated with an increased risk of MAS.
2.	Huang et al. [15]	2021	Taiwan	Case-control study/ Retrospective	Adults	39	780745 person-months	The incidence of death was higher in SLE patients with MAS as compared to all patients without MAS and age/sex/index year-matched (1:4) patients without a history of MAS. The incidence rate ratio (IRR) was 1.88 for mortality in SLE patients who developed MAS after their diagnosis as compared to SLE patients who did not develop MAS after diagnosis.
3.	Ahn et al. [16]	2017	South Korea	Retrospective analysis, Case-control	Adults	54	-	In febrile SLE patients, multivariate analysis showed that the in-hospital mortality rates were higher than those without MAS ( 13% in MAS as compared to 3% in patients without MAS). Univariate logistic regression analysis showed age, CRP, LFTS, RFTs, presence of MAS on admission, and MAS throughout hospital stay associated with in-hospital mortality
4.	Dall’ara et al. [17]	2018	Italy	Retrospective, Observational	Adults	7	54 months	Two ICU admissions, no in-hospital mortality were observed. One death 44 months after MAS but due to an unrelated cause.
5.	Takahashi et al. [18]	2014	Japan	Retrospective, Observational	Adults	7	-	Only one death out of seven patients (14%) was reported. Low CRP and high Hb levels were associated with response to steroid therapy while high ferritin levels and low leukocyte counts were associated with response to cyclosporine therapy.
6.	Fukaya et al. [19]	2008	Japan	Retrospective, Observational	Adults	18	85.6 months	Two in-hospital deaths were reported out of 18 SLE with MAS cases (11%). Univariate analysis showed age over 50, presence of infection, low CRP, low leukocyte, and platelet counts were related to mortality.
7.	Lambotte et al. [20]	2006	France	Retrospective, Observational	Adults	12	87.75 months	Five ICU admissions and no in-hospital mortality were reported in the 12 patients studied. One patient died after 15 months of diagnosis due to septic shock. four patients with SLE had recurrent episodes of MAS.
8.	Gavand et al. [21]	2017	France	Retrospective, Observational	Adults	89	42.4 months	4.9% in-hospital mortality and 32% ICU admissions were observed in 89 patients. In multivariate analysis, thrombocytopenia and high CRP levels are associated with increased risk of ICU admission.

**Table 4:** Table of features and trigger factors associated with MAS in patients with lupus *Studies were reported in form of median values rather than means **LDH, AST, and ALT levels were reported in terms of the upper limit of normal rather than actual mean values ***Hepatomegaly and splenomegaly were reported together rather than independently ˧ Study by Huang et al. discusses only mortality outcomes and association of SLE. Therefore, the other characteristics are not reported. SLE = systemic lupus erythematosus, MAS = macrophage activation syndrome, WBC = white blood cell count, Hb = hemoglobin, LDH = lactate dehydrogenase, AST = aspartate aminotransferase, ALT = alanine aminotransferase, CRP = C-reactive protein, ESR = erythrocyte sedimentation rate, C3 = complement component 3, C4 = complement component 4, ANA = antinuclear antibody, anti-dsDNA = anti-double-stranded DNA, CNS = central nervous system

Characteristics		Cohen et al. [14]	Huang et al.˧ [15]	Ahn et al. [16]	Dall'ara et al. [17]	Takahashi et al. [18]	Fukaya et al. [19]	Lambotte et al. [20]	Gavand et al. [21]
Age (Mean)		43	30-45	37	31.5	41.3	34.2	25.16	32
Sex (Female) n (%)		18 (78.3)	-	37 (68)	7 (100)	7 (100)	15 (83)	10 (83)	72 (81)
Duration of SLE (months)		65.7	-	1.9	-	-	85.2	-	108
New-onset SLE n (%)		4 (17)	-	35 (64)	7 (100)	5 (77)	1 (0.05)	9( 75)	41 (46)
Trigger Factors:	Flare	-	-	34.3%	7 (100)	7 (100)	16 (88)	8 (66)	68 (66)
	Infection	-	-	-	1 (14)	-	2 (12)	2 (16)	45 (43)
	Drugs	-	-	-	-	-	-	-	1 (.009)
Lab Characteristics		Cohen et al. [14]	Huang et al.˧ [15]	Ahn et al. [16]	Dall'ara et al. [17]	Takahashi et al. [18]	Fukaya et al. [19]	Lambotte et al. [20]	Gavand et al.* [21]
WBC (10^9^/L)		2.02	-	2.66	2.1	1.74 +/- 0.60	2.03	2 +/- 0.9	-
Hb (gm/L)		-	-	100	73	86 +/- 16	91.4	8.5 +/- 1.2	8.5
Platelets(10^9^/L)		41	-	106.0	132	82 +/- 51	73.6	98 +/- 61	93
LDH (U/L)		671.5	-	-	834	-	1080	4xN**	2.5xN**
AST(U/L)		161	-	100.5	282	-	194.6	7.3xN**	5.3xN**
ALT(U/L)		68	-	45	113	-	-	4.7xN**	-
CRP (mg/L)		60.5	-	10.3	47	33	47.54	15 +/-21	59
ESR (mm/hr)		72	-	42.5	24	-	-	-	-
Ferritin (µg/L)		8111	-	1833.5	6131	15491 +/- 12666	3357.6	8509 +/- 11,77	4717
Fibrinogen (g/L)		-	-	2.35	1.51	-	-	2.9 +/- 1.3	3.38
Triglycerides (mg/dl)		239	-	215	493	-	-	210 +/- 87.5	336
Low C3 n (%)		-	-	37	31	-	-	-	45 (56.2)
C4 n (%)		-	-	8.4	10	-	-	-	-
ANA n (%)		91.3	-		7 (100)	-	-	12 (100)	-
Anti-dsDNA n (%)		-	-	-	6 (85)	3(7)	-	10 (83)	52 (62.7)
Organ and System involvement		Cohen et al. [14] (n=23)	Huang et al. ˧ [15]	Ahn et al. [16] (n=54)	Dallara et al. [17] (n=7)	Takahashi et al. [18] (n=7)	Fukaya et al. [19] (n=18)	Lambotte et al. [20] (n=12)	Gavand et al. [21] (n=103)
Fever n (%)		-	-	-	7 (100)	-	17(94)	12 (100)	103 (100)
Arthritis n (%)		12 (52.2)	-	6 (11)	3 (43)	3(43)	-	6 (50)	38 (36.9)
Mucocutaneous Involvement n (%)		-	-		5 (71)	7(7)	-	6 (50)	44 (42.7)
Malar rash		13 (56.5)	-	17 (31)	-	-	-	2 (16)	-
Oral ulcers		6 (26.1)	-	5 (9)	-	-	-	-	-
Photosensitivity		7 (30.4)	-	3 (5)	-	-	-	-	-
Discoid lesions		5 (21.7)	-	-	-	-	-	-	-
Nephritis n (%)		17 (73.9)	-	21 (38)	0	4 (57)	-	5 (41)	3 (2.9)
Cardiac n (%)		-	-	-	-	4 (57)	-	7 (58)	24 (23.3)
Serositis n (%)		8 (34.8)	-	13 (24)	4 (57)	-	-	3 (25)	-
CNS involvement n (%)		6 (26.1)	-	4 (7)	1 (14)	5 (71)	7 (38)	2 (16)	38 (36)
Pulmonary involvement n (%)		-	-	-	-	5 (71)	-	4 (33)	15 (14.6)
Lymphadenopathy n (%)		-	-	-	7 (100)	-	4 (22)	8 (66)	-
Hepatomegaly n (%)		-	-	-	5(71)	5(7)***	-	2 (16)	38 (36.9)
Splenomegaly n (%)		-	-	-	5(71)	5(7)	8(44)	3(25)	28 (27.2)
Hematologic n (%)		21 (91.3)	-	46 (85)	7 (100)	7 (100)	16 (87)	12 (100)	-

Mortality Outcomes

All the studies showed that the number of ICU admissions, as well as mortality rates, were higher in patients who developed MAS secondary to lupus as evident in Table 3. Mortality rates were reportedly higher in American (Cohen et al.), South Korean (Ahn et al.), and Japanese studies (Takahashi et al. and Fukaya et al.) [14,16,18,19]. Studies done in Italy and France had lower mortality rates [17,20,21]. The factors identified in these studies that contributed to high ICU admissions and high mortality rates were age over 50, presence of infection, leukopenia, thrombocytopenia, high CRP, high liver function tests (LFTs), renal function tests (RFTs), presence of MAS on admission and MAS throughout the hospital stay [16,19,21].

Trigger Factors and Lab characteristics

The most identified trigger factor that led to the development of MAS in our review was lupus flare as shown in Table 4 which signifies that the flare itself is an independent risk factor for the development of MAS. This was followed by infections in patients with SLE.

Six studies [14,16-20] reported mean leukocyte counts and hemoglobin as low. Seven studies [14,16-21] reported thrombocytopenia. This again goes with the fact that cytopenias (bi- or pancytopenia) are a predominant feature in patients who developed MAS secondary to SLE.

Five studies [14,17,19-21] reported LDH levels which were in a range of two to four times the upper limit of normal. Aspartate aminotransferase levels were reported high in these studies but with considerable variation from 2.5 to 7.8 times the upper limit of normal [14,16,17,19-21].

Interestingly, reporting of CRP was highly variable. Ahn et al. [16] and Lambotte et al.’s [20] study showed low CRP values of 10 and 15 mg/L which contrasted with the remaining studies that reported CRP of 33 to 60.5, respectively. However, all these studies reported CRP less than 100 mg/L [14, 16-21]. Ferritin was reported above 1000s in all the studies [14, 16-21] except in the one by Takahashi et al. [18] that reported it in 10,000s. Unfortunately, most of our studies did not include low C3 and C4 levels to differentiate flares from infection as evident from Table 4.

Organ Involvement

Fever was the most consistent finding along with hematological involvement. arthritis was less frequently reported in these studies as reported in Table 4. Organ involvement in the remaining studies showed considerable variability among different studies.

Discussion

It has been well-known that autoimmune diseases can lead to the development of hemophagocytic lymphohistiocytosis (HLH), also known as macrophage activation syndrome (MAS). However, most of these AI diseases have been thought to predispose to HLH due to infectious or other underlying etiology rather than the disease itself [22-23]. Several studies have been done in this field regarding the cause of HLH in autoimmune diseases. Unfortunately, not many studies have dealt with individual diseases leading to HLH, the risk factors leading to it, and the outcomes associated with it. It is thought that HLH secondary to SLE, JIA, and Still’s disease is associated with disease flares rather than an underlying etiology [23]. Our review focused on identifying trigger factors leading to the development of HLH in the lupus population as well as identifying mortality outcomes and risk factors that increased mortality in this subset of patients. In addition, we also summarized patients’ clinical and laboratory characteristics and reported the features of various studies associated with this disease.

Epidemiology

Our review identified that most adult populations who had an episode of MAS fell into the third and fourth decades of life [14-21]. Also, it was evident that most SLE patients were females and MAS episodes were also observed in higher numbers in females except in Ahn et al.’s [16] study where 68% of episodes were reported in females but still a higher percentage (32%) were males. It is known that the female:male ratio in SLE patients is 10:1, but the female:male ratio of MAS in SLE patients in Ahn et al.'s study was 1.4:1 [1,16]. The combined total numbers of females were 166 to 44 males with a ratio of 3.77 to 1 in seven out of eight studies [14,16-21]; this may point towards males as being an independent risk factor that can lead to MAS in lupus patients. This also aligns with HLH due to other underlying causes (infections, malignancy, etc.) where there is a slightly high male preponderance [24-26]. This might indicate that males are in general at a higher risk of hemophagocytic syndromes than females. However, this relationship needs to be investigated further in larger studies.

Trigger Factors

Most studies identified flares as the most susceptible cause of HLH. Our review also supports the notion that flares are the leading cause of HLH in lupus patients followed by infections [16-21]. However, Takahashi et al. [18] focused only on SLE flares and excluded infections and drugs as potential aggravating factors of HLH. Still, the remaining studies did show flares and HLH development with new-onset SLE as major factors that could predispose to HLH. In addition, the corticosteroids and cyclosporin treatment in these studies showed an efficacious response that favored the underlying autoimmune etiology [16,17,19-21]. Few cases of drug-induced and one report each of malignancy-induced MAS and pregnancy-induced MAS were observed by Gavand et al. [21]. Though rare causes, they still seem to be important with several case reports identified related to them [27-29]. In addition, Gavand et al. [21] showed that viral infections seemed to be more prevalent in triggering HLH in lupus patients, with Epstein-Barr virus (EBV) being the most common trigger of infection, reported in 22/73 patients followed by cytomegalovirus (CMV). Bacteremia was observed in 16 cases, with S.aureus being the most prevalent micro-organism isolated, followed by E.coli [21].

In Cohen et al.’s [14] study, increasing SLEDAI scores were found to be a risk factor for MAS development in lupus patients, while hydroxychloroquine use and arthritis were reducing the risk of MAS. Intriguingly, arthritis was shown to be less common in this group of patients in our review. Except for Cohen et al., who claimed 52% of their patients had arthritis, five out of eight studies indicated less than 50% joint involvement [16-18,20,21]. This is significant because arthritis is the most common manifestation in patients with SLE, accounting for 84 to 90%. Arthritis was reportedly less frequently seen in patients who developed MAS [30].

Mortality Factors

All the studies showed that the number of ICU admissions, as well as mortality rates, were higher in patients who developed MAS secondary to lupus compared to lupus patients without MAS, as seen in Table 3. Mortality was reportedly around 4% to 19% in all these studies with Huang et al.’s [15] study showing an incidence rate ratio for mortality of 1.88 in SLE patients who had MAS than those who didn’t develop MAS. Overall, mortality rates in lupus patients were still less than mortality rates in patients who had hemophagocytic lymphohistiocytosis due to malignancy and infections [24].

Ahn et al. [16] used univariate logistic regression models to show that thrombocytopenia, low CRP levels, blood urea nitrogen (BUN) and creatinine (Cr) levels, total protein and albumin levels, LFTs, MAS on admission and throughout the hospital stay, were risk factors of in-hospital mortality. However, when multivariate regression was used, BUN levels and MAS throughout the hospital stay were the only significant factors contributing to the high in-hospital mortality rates.

There was a disparity to this in Fukaya et al.’s [19] study which used univariate analysis that indicated that older age of more than 50 years, presence of infection, leukopenia, thrombocytopenia, and high CRP levels were associated with mortality. This was further subjected to multivariate analysis which showed infection and high CRP levels were related to a poorer prognosis. This disparity might be due to the inclusion of both adult-onset Still’s disease (AOSD) and SLE patients in their analysis. Ahn et al.’s [16] study were also in contrast to Gavand et al.’s [21] study which included multivariate analysis of risk factors associated with high admission in ICU. This analysis identified high CRP and thrombocytopenia as only factors associated with a high risk of ICU admissions, again supporting Fukaya et al.’s [19] point of high CRP to be a poor prognostic factor.

All the above-mentioned studies identified similar risk factors to Birndt et al. [31] who analyzed risk factors of mortality via univariate and multivariate analysis in adult secondary HLH patients in general and found out that age over 50 years, neutropenia, thrombocytopenia, and low albumin levels were indicators of poor prognosis.

Lab Characteristics and Diagnostic Criteria

The lab parameters observed in these studies were as per the relevant diagnostic criteria according to the time these studies were published. It was evident in these studies that the HLH-2004 criteria were difficult to be fulfilled in most patients as time is the key for management and investigations like hemophagocytosis in bone marrow lack both specificity and sensitivity [14,16,21]. Also studies like soluble CD25, CXCL9, and natural killer (NK) cell activity although specific, are not possible in many centers of the world as of yet. Therefore, diagnostic criteria should be feasible to make early diagnostic decisions and interventions. Gavand et al.’s study showed 100% fulfillment of the 2016 diagnostic criteria for MAS due to JIA. In addition, they reported high levels of LDH (92.3%), AST (94.7%), ferritin (96%) and CRP (84.5%) [21].

Interestingly, reporting of mean CRP levels was highly variable. Ahn et al. [16] and Lambotte et al.’s [20] study showed low CRP values of 10 and 15 mg/L which contrasted with the remaining studies that reported CRP of 33 to 60.5, respectively. However, all these studies reported CRP less than 100 mg/L [14,16-21]. Ahn et al. [16] also reported low levels of CRP along with a rise in transaminases and high ferritin levels as factors linked with an increased risk of MAS. These low levels of CRP might be explained by flares being a predominant cause in Ahn et al's [16] study. This finding contradicted Cohen et al. [14] and Gavand et al.'s [21] findings of high CRP which might be linked to secondary infections causing an increase in CRP in MAS patients as compared to a lupus flare. Additional studies are needed for the monitoring of CRP levels secondary to flares or infections in MAS associated with SLE patients.

Mean ferritin levels were reported above 1000s in all the studies [14,16-21], except for Takahashi et al.’s [18] study which reported it in the 10,000s. This indicates that the cut-off for ferritin levels may be increased to 1000s to increase the specificity of JIA criteria as levels below 1000 may be non-specific. This finding was also supported by Assari et al.’s prospective study that dealt with pediatric autoimmune patients and found out that even levels above 5000s were needed to diagnose this condition [32]. Assari et al. also studied dynamic changes in the pediatric population and reported thrombocytopenia and falling platelet counts with a difference of >3000/µL as well as AST/ALT level changes as highly significant of early MAS [32]. Unfortunately, no studies available compared dynamic and static changes in the adult population. However, all the studies [14,16-21] reported in our review showed low platelet levels <150,000, signifying thrombocytopenia as an important feature in MAS in SLE patients differentiating it from flares along with high AST and LDH levels as mentioned in Table 4. Five out of eight studies described triglyceride (TG) levels, and in these studies TG levels were >200 mg/dl or moderately elevated [14,16,17,20,21]. Four out of eight studies mentioned fibrinogen levels [16,17,20,21]. Except for Dall'ara et al.'s [17] study which showed slightly low fibrinogen levels (1.51 g/L), the remaining studies showed normal fibrinogen levels. Nevertheless, this finding did confirm the importance of ferritin levels, LFTs, and TG levels in differentiating MAS from SLE flares. These findings were also in solidarity with Lin et al.’s study of lupus in the pediatric population and Li et al.’s study in adults with secondary HLH suggesting that there might not be a huge difference in pediatric and adult-onset secondary HLH [33,34]. However, the cutoffs for HLH due to various causes may need further studies.

Organ Involvement

Various degrees of organ involvement can be seen in Table 4. Fever was a common feature in four out of eight studies [17,19-21]. Almost all the patients in these studies developed a fever that met the HLH-2004 diagnostic criteria. Arthritis, which is a common symptom in patients with SLE, was seen in surprisingly few patients who had SLE and MAS, as indicated in Table 4. In addition, the majority of the studies revealed a significant level of hematological involvement. Nephritis which was reported in six out of eight studies listed above in Table 4 was reported in higher numbers by Cohen et al. [14] at 73.9% but surprisingly was a rare feature in Dall'ara et al. [17] and Gavand et al.'s studies [21]. Lambotte et al.’s [20] study was unusual as it reported a higher number of cardiological involvement (58%) more than arthritis and mucocutaneous involvement which are predominant features in patients who have SLE alone [30]. In contrast, only 23.3% of MAS with SLE patients had cardiac complications in Gavand et al.’s study [21]. In short, fever and hematological involvement were the most reported and consistent features in all the studies contrary to the considerable variability in reporting of features in the remaining studies.

Limitations

One of the major limitations of this review was that all studies done were retrospective. This might be because hemophagocytic syndrome is a rare complication of SLE and might be under-recognized and under-reported due to its similarity with lupus flares or superinfections itself. Another limitation is the fact that multiple criteria over the years have been used to diagnose systemic lupus erythematosus and macrophage activation syndromes that can lead to heterogeneity of studies. In addition, the validation of these criteria for diagnosis of MAS in SLE is still to be done with specificity and sensitivity to be determined.

## Conclusions

Macrophage activation syndrome secondary to systemic lupus erythematosus despite being a rare entity is associated with higher rates of ICU admissions and in-hospital mortality especially due to the diagnostic challenges related to this syndrome. The fact that both conditions mimic each other, can lead to a delayed diagnosis and interfere with timely treatment. Special considerations for patients with hematological involvement in SLE, the elderly, and those with higher ferritin levels should be made. This may include workup of MAS and early intervention as these groups are associated with higher complication risks. In addition, criteria for HLH should be modified for different causes (infections, autoimmune, and malignancies) to account for its heterogeneity in presentation.

## Appendices

Appendix A

Phase 1

*Topic: HLH in SLE    *                                                                                                                                                                                                                                                                Research Question: To identify risk factors (genetic or environmental) leading to HLH in patients with SLE

PICO

Population/Problem: all patients with SLE who had at least 1 episode of HLH

Intervention: No specific interventions or exposures were used in the study

Comparisons/Control: No specific comparison or controls were used in the study

Outcome

1. Risk factors leading to HLH in patients with SLE

2. Factors leading to increased mortality in these patients

3. Clinical characteristics of patients in the study

Phase 2

Search Strategy

Eligibility criteria: All patients with systemic lupus erythematosus who had at least one episode of hemophagocytic lymphohistiocytosis will be included in the review. Patients should fulfill any one of the three criteria of SLE, namely the ACR criteria, 2012 Systemic Lupus International Collaborating Clinics (SLICC) criteria for diagnosis of SLE, or 2019 criteria and HLH criteria for diagnosis of HLH. All articles that are peer-reviewed, free full-text articles in the English language will be included. All articles from January 2001 to May 2021 will be included. All cross-sectional cohort studies and trials were included in the study to identify potential risk factors leading to HLH in patients with SLE and to identify mortality risk factors and outcomes. We also used descriptive studies to identify specific features of the adult lupus population who suffered from MAS.

We excluded all patients with SLE without HLH development. For this review, we also excluded the pediatric population. All editorials, case reports, and animal studies were omitted. Furthermore, we also excluded all articles that were not published in the last 20 years, were in languages other than English, were unpublished, and articles in gray literature. We removed all studies in which mortality outcomes or risk factors leading to HLH in SLE were not specified.

Data Collection

Concepts: (1) HLH and (2) SLE

Keywords: Hemophagocytic syndromes, hemophagocytic lymphohistiocytosis, secondary hemophagocytic lymphohistiocytosis, macrophage activation syndrome, HLH, Systemic lupus erythematosus, Lupus erythematosus, lupus

MeSH keywords: ((((("Lupus Erythematosus, Systemic"[Majr]) OR "Lupus Erythematosus, Systemic"[Mesh:NoExp]) AND "Lymphohistiocytosis, Hemophagocytic"[Majr]) OR "Lymphohistiocytosis, Hemophagocytic"[Mesh:NoExp]) AND "Macrophage Activation Syndrome"[Majr]) OR "Macrophage Activation Syndrome"[Mesh:NoExp] SLE:(( "Lupus Erythematosus, Systemic/etiology"[Majr] OR "Lupus Erythematosus, Systemic/genetics"[Majr] )) OR ( "Lupus Erythematosus, Systemic/etiology"[Mesh:NoExp] OR "Lupus Erythematosus, Systemic/genetics"[Mesh:NoExp] )Hemophagocytic lymphohistiocytosis:("Lymphohistiocytosis, Hemophagocytic"[Majr]) OR "Lymphohistiocytosis, Hemophagocytic"[Mesh:NoExp] ("Macrophage Activation Syndrome"[Majr]) OR "Macrophage Activation Syndrome"[Majr:NoExp]

Combined keywords: hemophagocytic syndromes OR hemophagocytic lymphohistiocytosis OR macrophage activation syndrome OR HLH OR secondary hemophagocytic lymphohistiocytosis AND systemic lupus erythematosus OR SLE ((((("Lupus Erythematosus, Systemic"[Majr]) OR "Lupus Erythematosus, Systemic"[Mesh:NoExp]) AND "Lymphohistiocytosis, Hemophagocytic"[Majr]) OR "Lymphohistiocytosis, Hemophagocytic"[Mesh:NoExp]) AND "Macrophage Activation Syndrome"[Majr]) OR "Macrophage Activation Syndrome"[Mesh:NoExp]

**Table 5:** Results from the search of PubMed, ScienceDirect, Cochrane library, ProQuest, and Google Scholar databases

Keywords	Database	Total Articles
hemophagocytic syndromes OR hemophagocytic lymphohistiocytosis OR macrophage activation syndrome OR HLH OR secondary hemophagocytic lymphohistiocytosis	PubMed ( Advanced search)	9182
systemic lupus erythematosus OR SLE	PubMed ( Advanced search)	79,769
hemophagocytic syndromes OR hemophagocytic lymphohistiocytosis OR macrophage activation syndrome OR HLH OR secondary hemophagocytic lymphohistiocytosis AND systemic lupus erythematosus OR SLE Filters: Free full text	PubMed ( Advanced search)	12,338
hemophagocytic syndromes OR hemophagocytic lymphohistiocytosis OR macrophage activation syndrome OR HLH OR secondary hemophagocytic lymphohistiocytosis AND systemic lupus erythematosus OR SLE	PubMed ( Advanced search)	36,261 results
hemophagocytic syndromes OR hemophagocytic lymphohistiocytosis OR macrophage activation syndrome OR HLH OR secondary hemophagocytic lymphohistiocytosis AND systemic lupus erythematosus OR SLE ((((("Lupus Erythematosus, Systemic"[Majr]) OR "Lupus Erythematosus, Systemic"[Mesh:NoExp]) AND "Lymphohistiocytosis, Hemophagocytic"[Majr]) OR "Lymphohistiocytosis, Hemophagocytic"[Mesh:NoExp]) AND "Macrophage Activation Syndrome"[Majr]) OR "Macrophage Activation Syndrome"[Mesh:NoExp]	PubMed Combined keywords	513 results
	PubMed final selected	108
hemophagocytic lymphohistiocytosis and systemic lupus erythematosus	Google Scholar	4160
hemophagocytic syndromes OR hemophagocytic lymphohistiocytosis OR macrophage activation syndrome OR HLH OR secondary hemophagocytic lymphohistiocytosis AND systemic lupus erythematosus OR SLE	Google Scholar	3280 (192)
	Google Scholar final	167
hemophagocytic syndromes OR hemophagocytic lymphohistiocytosis OR macrophage activation syndrome OR HLH OR secondary hemophagocytic lymphohistiocytosis AND systemic lupus erythematosus OR SLE	ScienceDirect	3338
hemophagocytic syndromes OR hemophagocytic lymphohistiocytosis OR macrophage activation syndrome OR HLH OR secondary hemophagocytic lymphohistiocytosis AND systemic lupus erythematosus OR SLE	ScienceDirect	final 21
hemophagocytic syndromes OR hemophagocytic lymphohistiocytosis OR macrophage activation syndrome OR HLH OR secondary hemophagocytic lymphohistiocytosis AND systemic lupus erythematosus OR SLE	Cochrane library	115
Hemophagocytic syndromes OR hemophagocytic lymphohistiocytosis OR macrophage activation syndrome OR HLH OR secondary hemophagocytic lymphohistiocytosis AND systemic lupus erythematosus OR SLE	ProQuest	248
	ProQuest final selected	5
	Duplicates removed	26

	Author	Title	Year	Type of Study	Population	Sample Size	Outcome
1	Yu et al.	Outcomes and prognostic factors associated with 180-day mortality in Taiwanese pediatric patients with Hemophagocytic Lymphohistiocytosis	2020	Retrospective study	Pediatric	3 SLE pts	
2	Santos et al.	Hemophagocytic lymphohistiocytosis: a case series analysis in a pediatric hospital	2021	Case series, retrospective	Pediatric	6 SLE pts	
3	Zou et al.	Clinical and laboratory features, treatment, and outcomes of macrophage activation syndrome in 80 children: a multi‑center study in China	2019	Cohort study	Pediatric	9 SLE pts	
4	Nishino et al.	Usefulness of soluble CD163 as a biomarker for macrophage activation syndrome associated with systemic lupus erythematosus	2019	Cohort study	N/S	17 SLE pts	
5	Lorenz et al.	Adult macrophage activation syndrome–haemophagocytic lymphohistiocytosis: ‘of plasma exchange and immunosuppressive escalation strategies’ – a single centre reflection	2020	Retrospective study	Adults	4 SLE pts	
6	Maruyama et al.	Cytokine Profiles of Macrophage Activation Syndrome Associated with Rheumatic Diseases	2010	Retrospective study	8 Adults 1 Peds	9 SLE pts	
7	Parodi et al.	Macrophage Activation Syndrome in Juvenile Systemic Lupus Erythematosus A Multinational Multicenter Study of Thirty-Eight Patients	2009	Case control study	Peds	38 SLE	
9	Cohen et al.	Arthritis and use of hydroxychloroquine associated with a decreased risk of macrophage activation syndrome among adult patients hospitalized with systemic lupus erythematosus	2018	Case control study	Adults	23 SLE	
10	Huang et al.	Bidirectional association between systemic lupus erythematosus and macrophage activation syndrome: a nationwide population-based study	2021	Case control study, retrospective	Adults	39 SLE	
11	Li X et al.	Clinical features of macrophage activation syndrome in the adult northern Chinese population	2014	Retrospective	Adults	2 SLE	
12	Guo Y et al.	Clinical features and prognostic factors of adult secondary hemophagocytic syndrome Analysis of 47 cases	2017	Retrospective	Adults	8	
13	Gavand et al.	Clinical spectrum and therapeutic management of systemic lupus erythematosus-associated macrophage activation syndrome: A study of 103 episodes in 89 adult patients	2017	Retrospective	Adults	89	
14	Obayo et al.	Adult secondary hemophagocytic lymphohistiocytosis	2021	Case series	Adults	2	
15	Ahn et al.	In-hospital mortalityinfebrilelupuspatientsbasedon2016 EULAR/ACR/PRINTOclassification criteria for macrophage activation syndrome	2017	Retrospective	N/S	54	
16	Lin et al.	Clinical analysis of macrophage activation syndrome in pediatric patients with autoimmune diseases	2012	Retrospective	Peds	2 SLE	
17	Gormezano et al.	Macrophage activation syndrome: A severe and frequent manifestation of acute pancreatitis in 362 childhood-onset compared to 1830 adult-onset systemic lupus erythematosus patients	2016	Retrospective	Adults+Peds	17 SLE+AP+MAS	
18	Dallara et al.	Macrophage activation syndrome in adult systemic lupus erythematosus: report of seven adult cases from a single Italian rheumatology center	2018	Retrospective	Adults	7 SLE	
19	Apodaca et al.	Prognostic Factors and Outcomes in Adults With Secondary Hemophagocytic Lymphohistiocytosis: A Single-center Experience	2018	Retrospective analysis	Adults	3 SLE	
20	Hansen et al.	Ruxolitinib as adjunctive therapy for secondary hemophagocytic lymphohistiocytosis: A case series	2020	Case series	Adult	1 SLE	
21	Asra et al.	Ruxolitinib in adult patients with secondary haemophagocytic lymphohistiocytosis: an open-label, single-centre, pilot trial	2019	Cohort study	Adult	1 SLE	
22	Dubuc et al.	Secondary Macrophage Activation Syndrome Due to Autoimmune, Hematologic, Infectious and Oncologic Diseases. Thirteen Case Series and Review of the Literature	2014	Case series	Adult	2 SLE	
23	Tabata et al.	Hemophagocytic syndrome in elderly patients with underlying autoimmune diseases	2009	Retrospective study	Adult	2 SLE	
24	Ueda et al.	Refractory hemophagocytic syndrome in systemic lupus erythematosus successfully treated with intermittent intravenous cyclophosphamide: three case reports and literature review	2013	Case series	Adult	3 SLE	
25	Gupta et al.	Unusual Association of Hemophagocytic Lymphohistiocytosis in Systemic Lupus Erythematosus: Cases Reported at Tertiary Care Center	2016	Case series	Adult? Peds	2 SLE	
26	Wakabayashi et al.	Serum β2-microglobulin level is a useful indicator of disease activity and hemophagocytic syndrome complication in systemic lupus erythematosus and adult-onset Still’s disease	2013	Retrospective study	Adults	7 SLE	
27	Takahashi et al.	Predictors of the response to treatment in acute lupus hemophagocytic syndrome	2014	Retrospective study	Adults	7 SLE	
28	Campos et al.	Acute pancreatitis in juvenile systemic lupus erythematosus: a manifestation of macrophage activation syndrome?	2010	Retrospective study	Peds	11 SLE	
29	Fukaya et al.	Clinical features of haemophagocytic syndrome in patients with systemic autoimmune diseases: analysis of 30 cases	2008	Retrospective	Adults	8 pts	
30	Lambotte et al.	Characteristics and Long-Term Outcome of 15 Episodes of Systemic Lupus Erythematosus-Associated Hemophagocytic Syndrome	2006	Retrospective	Adults	15 pts	
